# Fully Integrated Low-Noise Readout Circuit with Automatic Offset Cancellation Loop for Capacitive Microsensors

**DOI:** 10.3390/s151026009

**Published:** 2015-10-14

**Authors:** Haryong Song, Yunjong Park, Hyungseup Kim, Dong-il Dan Cho, Hyoungho Ko

**Affiliations:** 1 Department of Electronics, Chungnam National University, Daejeon 305-764, Korea; E-Mails: zealshr@cnu.ac.kr (H.S.); pyjj90@cnu.ac.kr (Y.P.); hanafos24@cnu.ac.kr (H.K.); 2 ASRI/ISRC, Department of Electrical Engineering and Computer Science, Seoul National University, Seoul 151-744, Korea; E-Mail: dicho@snu.ac.kr

**Keywords:** capacitive sensing circuit, automatic offset cancellation loop (AOCL), correlated double sampling (CDS), capacitive microsensor

## Abstract

Capacitive sensing schemes are widely used for various microsensors; however, such microsensors suffer from severe parasitic capacitance problems. This paper presents a fully integrated low-noise readout circuit with automatic offset cancellation loop (AOCL) for capacitive microsensors. The output offsets of the capacitive sensing chain due to the parasitic capacitances and process variations are automatically removed using AOCL. The AOCL generates electrically equivalent offset capacitance and enables charge-domain fine calibration using a 10-bit R-2R digital-to-analog converter, charge-transfer switches, and a charge-storing capacitor. The AOCL cancels the unwanted offset by binary-search algorithm based on 10-bit successive approximation register (SAR) logic. The chip is implemented using 0.18 μm complementary metal-oxide-semiconductor (CMOS) process with an active area of 1.76 mm^2^. The power consumption is 220 μW with 3.3 V supply. The input parasitic capacitances within the range of −250 fF to 250 fF can be cancelled out automatically, and the required calibration time is lower than 10 ms.

## 1. Introduction

Capacitive microsensors based on microelectromechanical system (MEMS) technologies are used in various application areas, including accelerometers, gyroscopes, pressure sensors, touch-screen sensors, proximity sensors, and so on because of their small form factor, low-power characteristics, good temperature dependency, and low cost [1,2,3,4,5]. The capacitive sensing method, however, suffers from severe parasitic capacitance mismatches. The parasitic capacitance mismatches are often much higher (on the order of several hundreds of femtofarads to several picofarads) than sensing capacitance charges (on the order of several tens of femtofarads). The parasitic capacitance mismatches and random process variations result in large output offset variations, and in worst case, output saturation to VDD or GND. Thus, additional offset calibration steps are required using external calibration equipment. Moreover, to calibrate the output offset of the physical sensors such as accelerometer, special equipment with physical stimulus capability, such as a vibration exciter, is required. These physical calibration steps increase the production cost of the sensor system. For example, in case of BMC050 six-axis combo motion sensor of Bosch Sensortec, test and packaging cost is 35% of the production cost [6]. The on-chip automatic offset calibration circuit using binary-weighted capacitive digital-to-analog converter was reported [7]. In the previous research [7], the calibration resolution of the offset calibrator is limited by the minimum design rules, thus, accurate automatic offset calibration with sub-femtofarads resolution was not implemented.

This paper presents a fully integrated low-noise capacitive sensing circuit with automatic offset calibration loop (AOCL). The output offsets of the capacitive sensing chain due to the parasitic capacitances and process variations are automatically removed using the automatic offset cancellation loop (AOCL). The AOCL generates an electrically equivalent offset capacitance, and it enables charge-domain fine calibration using a 10-bit R-2R digital-to-analog converter, charge-transfer switches and a charge-storing capacitor. The presented circuit enables the automatic offset calibration for capacitive sensors without using external test equipment.

## 2. Circuit Description 

### 2.1. Top Level Architecture

**Figure 1:**
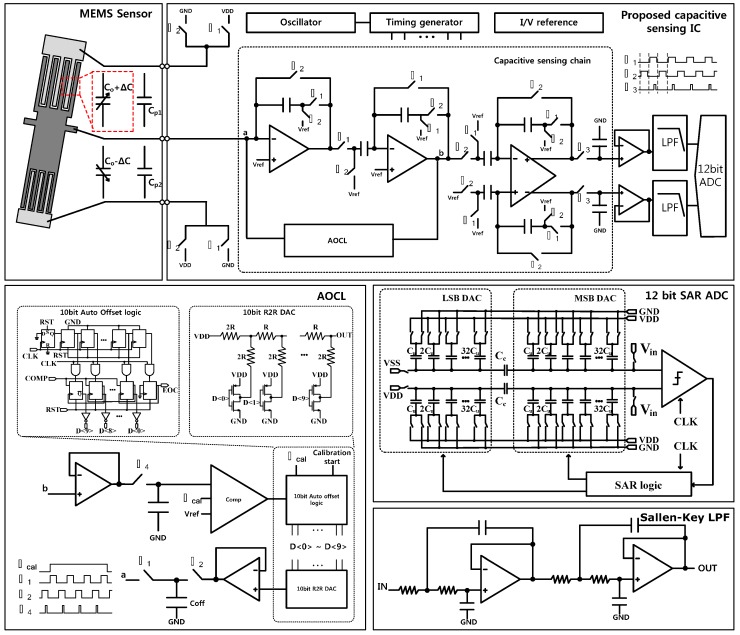
The block diagram of the proposed capacitive sensing circuit.

Figure 1 shows a block diagram of the proposed capacitive sensing circuit. The capacitive sensing chain adopts the correlated double sampling (CDS) technique to reduce the low frequency noise, including the 1/f noise [8,9,10]. The capacitive sensing chain is composed of three amplification stages. In the first stage, a capacitive sensing amplifier (CSA) converts the input capacitance change from MEMS sensor to output voltage. In the second stage, a programmable gain amplifier (PGA) amplifies the signal by 30 dB. The single-to-differential amplifier (SDA) of third stage converts the single-ended output of the PGA into a differential signal. The differential signals are converted to digital signals using a 12-bit successive approximation register (SAR) analog-to-digital converter (ADC) [11] after low pass filters and buffers. The 4th order Bessel low pass filter is implemented using continuous-time Sallen-Key active filter topology. The cut-off frequency of the low pass filter is 400 Hz. The AOCL adjusts the output offset to the desired value using binary search algorithm [12], and is implemented using a comparator, SAR logic, R-2R digital-to-analog converter (DAC), charge transfer switches, and a charge-storing capacitor.

### 2.2. Capacitive Sensing Amplifier (CSA)

**Figure 2:**
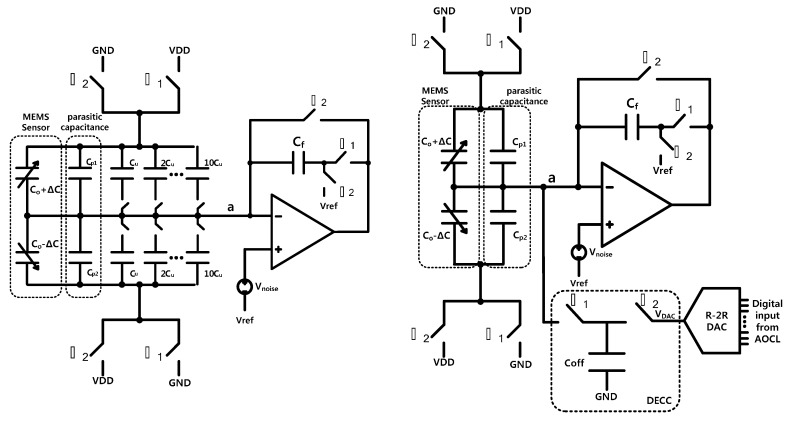
Capacitive sensing amplifier, (**a**) Conventional capacitor arrays calibration; (**b**) Charge-domain calibration.

Figure 2a,b show a correlated double sampled (CDS) CSA with offset calibration using conventional capacitor arrays and charge-domain fine calibration, respectively. The CDS operation removes unwanted low frequency noise components including flicker noise and DC offset. In conventional CSA, as shown in Figure 2a, binary-weighted capacitor arrays are used to cancel the input parasitic capacitance [13]. The resolution of conventional offset calibration scheme using capacitor arrays is limited by the minimum design rules of capacitors. For example, the minimum allowable capacitance is typically 16 fF (4 μm × 4 μm × 1 fF/μm^2^) for a general metal-insulator-metal (MIM) capacitor. Assuming a sensing capacitance of several femtofarads, to achieve the high offset accuracy, offset calibrations with sub-fF steps are required. In this design, to implement a calibration capacitance smaller than the physical design rule, charge-domain fine calibration scheme is implemented, as shown in Figure 2b [13]. The comparisons between the conventional capacitor array calibration scheme and the charge-domain calibration scheme are summarized in Table 1. The output voltage using charge-domain calibration (V_o_) and charge-domain equivalent capacitance (C_eq_) can be expressed as Equation (1). In this design, the charge storing capacitor, *C_off_*, is 250 fF, and the 10-bit R-2R DAC generates voltage from GND to VDD in 1024 steps. Thus, the charge-domain calibration circuit can generate an electrically equivalent offset capacitance in the range of ±250 fF with 0.488 fF step. The digital inputs of R-2R DAC are automatically determined by binary search algorithm of AOCL.

(1) $$\begin{array}{c} V_{o}=-\frac{V_{DD}(2\Delta C+(C_{p1}-C_{p2})+C_{eq})}{C_{f}}+V_{ref}\\ C_{eq}=\frac{\left(V_{ref}-V_{DAC}\right)}{V_{DD}}\times C_{off} \end{array}$$

**Table 1:** Comparisons between capacitor arrays calibration and charge-domain calibration.

Capacitor Implementation	Capacitor Arrays Calibration	Charge-Domain Calibration
	Physical Capacitor (MIM or PIP)	Electrically Equivalent Capacitor
Minimum capacitor	Limited by physical design rules (in this design, 16 fF = 4 μm × 4 μm × 1 fF/μm^2^)	LSB voltage × C_off_/V_DD_ (in this design, 0.488 fF = 1/1024 × 3.3/3.3 × 500 fF)
Size	Large (binary-weighted capacitor array)	Small (R-2R DAC, switches, and a charge-storing capacitor)
DC current	0	DC current consumption in R-2R DAC (1.2 μA in this design)

### 2.3. Programmable Gain Amplifier (PGA) and Single-to-Differential Amplifier (SDA)

**Figure 3:**
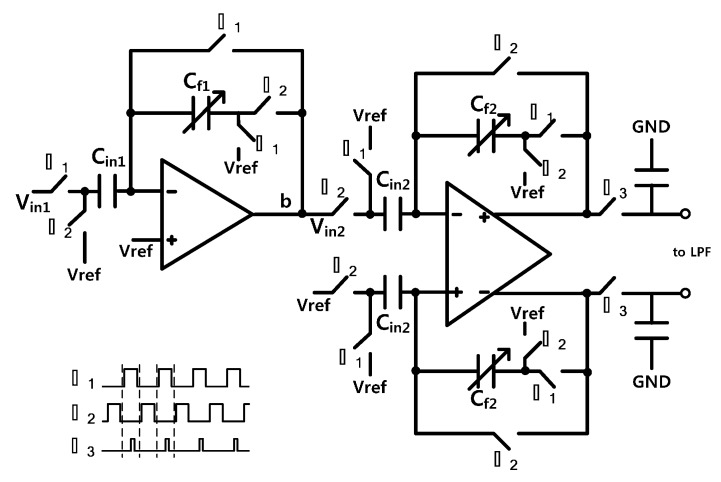
Programmable gain amplifier (PGA) and single to differential amplifier (SDA) circuit.

Figure 3 shows the PGA and SDA circuit. The PGA and SDA employ correlated double sampling technique to reduce the low-noise components. The Φ1 and Φ2 are non-overlapping clocks. In PGA, an input coupling capacitor (C_in1_) stores the input voltage signal and noise in Φ1 phase, and the stored charge is dumped in a feedback capacitor in Φ2 phase. The gain of PGA can be adjusted from 0 dB to 30 dB by programming C_f1_, as expressed in Equation (2). SDA works with the opposite phase of PGA, and the differential gain of SDA can be adjusted from 0 dB to 36 dB by programming C_f2_, as expressed in Equation (3). The output signal from the SDA is sampled on Φ3 phase.

(2) $$V_{PGA}=V_{ref}+V_{in1}\frac{C_{f1}}{C_{in1}}$$

(3) $$V_{S2D}=V_{ref}+2V_{in2}\frac{C_{f2}}{C_{in2}}$$

### 2.4. Automatic Offset Cancellation Loop (AOCL)

**Figure 4:**
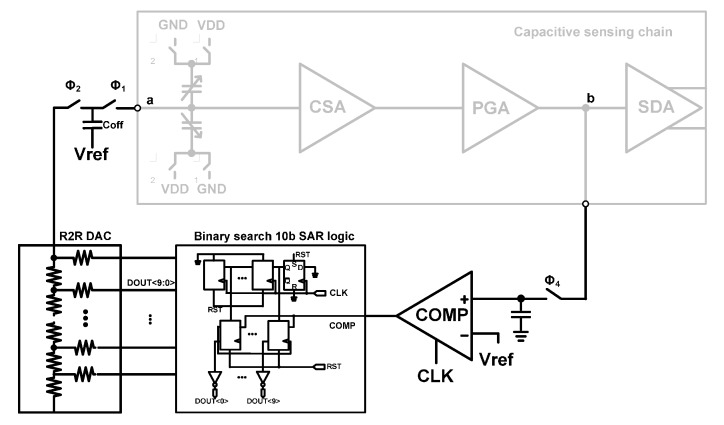
The proposed automatic offset cancellation loop (AOCL).

The proposed AOCL circuit is shown in Figure 4. The output voltage of PGA is sampled in Φ4 phase. In the 10-bit binary search successive approximation register (SAR) logic, the shift registers in the first row sequentially point towards the registers in the second row, which are to be updated to the comparator output. The registers (D-flip-flops) in the second row of the SAR logic become the digital control input of the R-2R digital to analog converter (DAC). 

**Figure 5:**
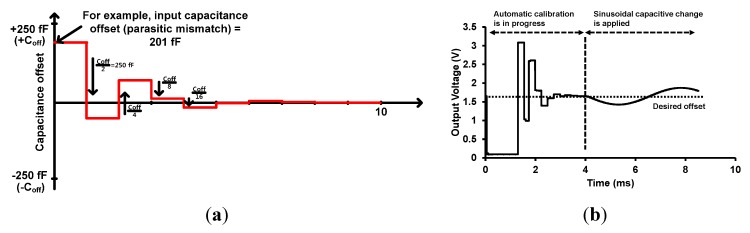
Operation example and simulation results of automatic offset cancellation loop (AOCL), (**a**) Operation example of AOCL; (**b**) Simulation result of AOCL.

An operation example of AOCL is illustrated in Figure 5a, and the steps involved in the operation are as follows. The sampled voltage is compared to Vref (desired voltage, VDD/2 = 3.3 V/2 = 1.65 V). Initially the MSB of DOUT<9:0> is “H”, and the initial value of DOUT<9:0> is “1,000,000,000”. With this initial value, the sampled PGA output and Vref are compared. If PGA output is higher than Vref, the MSB of DOUT<9:0> remains “H”, and DOUT<9:0> becomes “1,000,000,000”. If PGA output is lower than Vref, the MSB of DOUT<9:0> becomes “L”, and DOUT<9:0> becomes “0000000000”. DOUT<9:0> determines the digital input of R-2R DAC. The R-2R DAC, charge transfer switches, and charge storing capacitor generate an electrically equivalent offset capacitance. Next, the second bit of DOUT<9:0> is set to “H”, and DOUT<9:0> becomes “C100,000,000”, where C means the output of comparator. The PGA output is compared to Vref again, and the second bit of DOUT<9:0> is updated with new comparator output. After ten comparison cycles, the PGA output tracks the desired output of Vref, as shown in Figure 5b. The AOCL can calibrate the offset due to the input capacitive mismatches of MEMS sensors from −250 fF to 250 fF with 1024 steps.

## 3. Measurement Results

**Figure 6:**
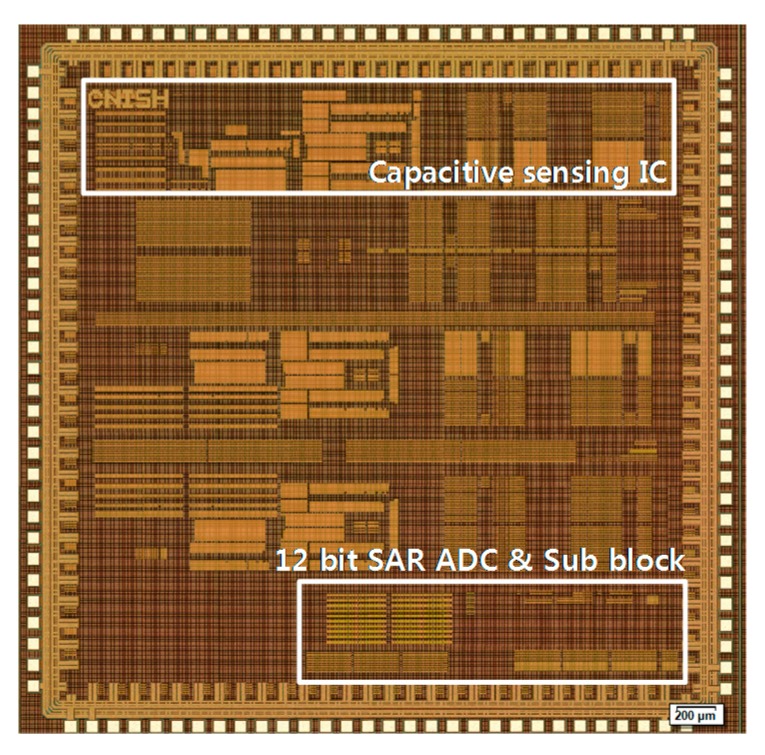
Chip micrograph.

**Figure 7:**
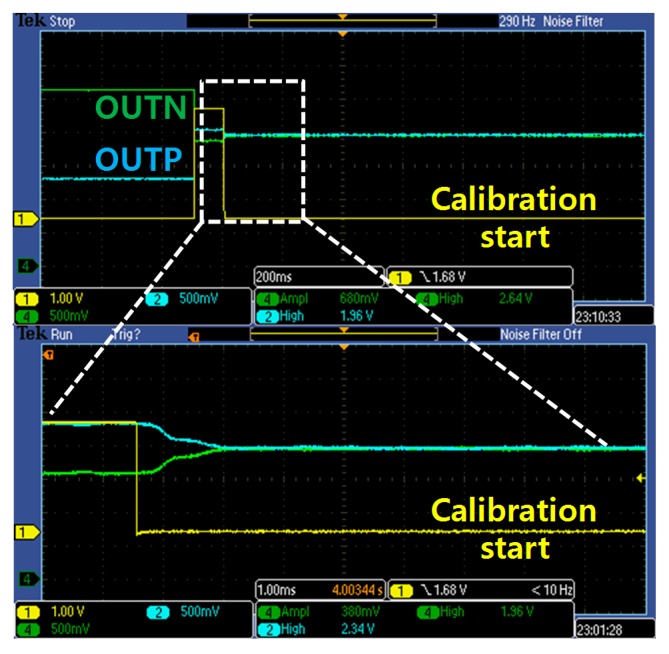
Measured analog output waveform with AOCL operation.

A die-photograph of the fabricated capacitive sensing IC is shown in Figure 6. The IC is fabricated using a 0.18 μm single-polysilicon six-metal complementary metal-oxide-semiconductor (CMOS) process with an active area of 1.76 mm^2^. Figure 7 shows the measured analog output waveform with the AOCL operation. The green line and blue line show the differential output of the low pass filter. The yellow line is enable signal of AOCL, and the AOCL is activated at the falling edge of enable signal. After the operation of AOCL, the initial offset is removed automatically. The offset calibration time is 10 ms with 1 kHz calibration clock. Because the AOCL is operated before the low pass filter, the calibration clock for AOCL can be faster than cut-off frequency of the low pass filter. The frequency of calibration clock and cut-off frequency of the low pass filter are also programmable. The default cut-off frequency of the low pass filter is 400 Hz.

**Figure 8:**
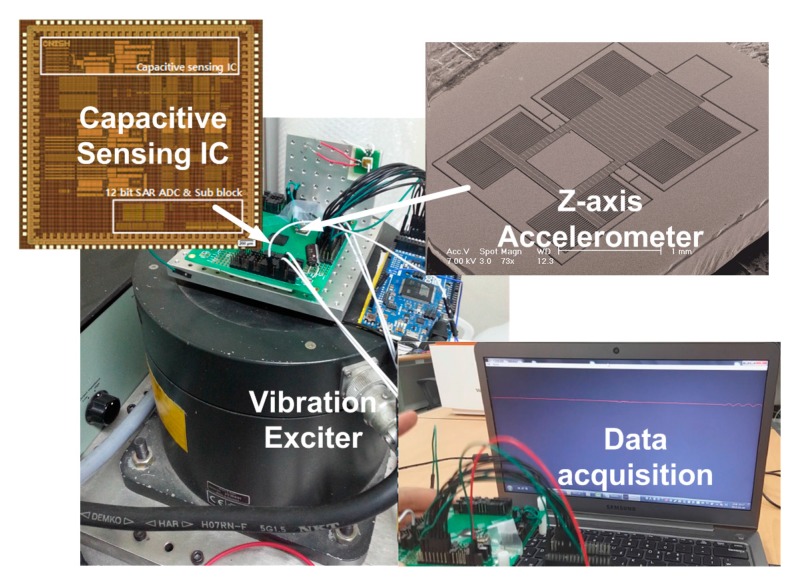
Measurement setup with capacitive accelerometer.

To evaluate the performance of the fabricated IC, the IC and MEMS *Z*-axis capacitive accelerometer are mounted on the vibration exciter, as shown in Figure 8. The detailed design and specification of the Z-axis accelerometer is described in [14].

**Figure 9:**
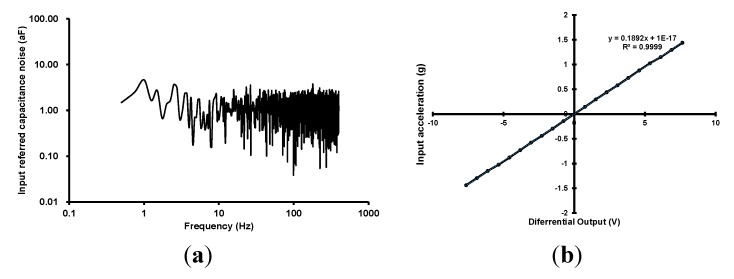
Measurement results, (**a**) Input-referred noise; (**b**) Input-output characteristics.

The measurement results are presented in Figure 9. Figure 9a shows the input referred noise spectrum. The input referred capacitance noise density, and integrated noise with 400 Hz bandwidth are 1.275 aF/√Hz and 25.5 aF_RMS_, respectively. The input-output characteristics with MEMS *Z*-axis accelerometer are presented in Figure 9b. The scale factor, input range and, non-linearity are 0.1892 V/g, ±7.5 g, and 0.81% FSO, respectively.

## 4. Conclusions

A fully integrated low-noise capacitive readout circuit with automatic offset cancellation loop (AOCL) for capacitive microsensors has been presented. The output offsets of the capacitive sensing chain due to the parasitic capacitances and process variations were automatically removed using the AOCL. The AOCL cancelled the unwanted offset by binary-search algorithm based on 10-bit SAR logic and charge-domain calibration circuits. The chip was implemented using 0.18 μm 1P6M CMOS process with an active area of 1.76 mm^2^. The power consumption was 220 μW with 3.3 V supply. The input parasitic capacitances within the range of −250 fF to 250 fF were cancelled out automatically, and the required calibration time was lower than 10 ms. The input referred capacitance noise density and integrated noise with 400 Hz bandwidth were 1.275 aF/√Hz and 25.5 aF_RMS_, respectively. The scale factor, input range, and non-linearity were 0.1892 V/g, ±7.5 g, and 0.81% FSO, respectively. The presented circuit enabled the automatic offset calibration for capacitive sensors without using external test equipment, and enabled low-cost manufacturing of the capacitive sensors.
